# Advances in the Microbiological Diagnosis of Prosthetic Joint Infections

**DOI:** 10.3390/diagnostics13040809

**Published:** 2023-02-20

**Authors:** Maria Eugenia Portillo, Ignacio Sancho

**Affiliations:** 1 Department of Clinical Microbiology, University Hospital of Navarra, Institute of Healthcare Research of Navarra IdiSNA, 31008 Pamplona, Spain; 2 Department of Orthopedics and Trauma Surgery, University Hospital of Navarra, 31008 Pamplona, Spain

**Keywords:** next-generation sequencing, non-culture methods, polymerase chain reaction, prosthetic joint infections

## Abstract

A significant number of prosthetic joint infections (PJI) are culture-negative and/or misinterpreted as aseptic failures in spite of the correct implementation of diagnostic culture techniques, such as tissue sample processing in a bead mill, prolonged incubation time, or sonication of removed implants. Misinterpretation may lead to unnecessary surgery and needless antimicrobial treatment. The diagnostic value of non-culture techniques has been investigated in synovial fluid, periprosthetic tissues, and sonication fluid. Different feasible improvements, such as real-time technology, automated systems and commercial kits are now available to support microbiologists. In this review, we describe non-culture techniques based on nucleic acid amplification and sequencing methods. Polymerase chain reaction (PCR) is a frequently used technique in most microbiology laboratories which allows the detection of a nucleic acid fragment by sequence amplification. Different PCR types can be used to diagnose PJI, each one requiring the selection of appropriate primers. Henceforward, thanks to the reduced cost of sequencing and the availability of next-generation sequencing (NGS), it will be possible to identify the whole pathogen genome sequence and, additionally, to detect all the pathogen sequences present in the joint. Although these new techniques have proved helpful, strict conditions need to be observed in order to detect fastidious microorganisms and rule out contaminants. Specialized microbiologists should assist clinicians in interpreting the result of the analyses at interdisciplinary meetings. New technologies will gradually be made available to improve the etiologic diagnoses of PJI, which will remain an important cornerstone of treatment. Strong collaboration among all specialists involved is essential for the correct diagnosis of PJI.

## 1. Introduction

Joint replacement surgery has been considered the best option to restore damaged joints, reduce pain, enhance joint function, and improve quality of life [1,2]. The use of implanted devices has therefore become widespread given their beneficial effect on quality of life, and in some cases, on patient survival rates [3]. The number of joint replacement surgeries is expected to continuously increase due to the aging of the population [4]. However, bearing an artificial joint produces wear, friction, and consequently, surface damage, eventually leading to prosthetic failure due to repetitive contact stresses. In fact, prosthetic survival is generally limited to about 15 years [1]. Moreover, prostheses can be associated with a variety of complications, such as infection.

Although prosthetic joint infection (PJI) occurs less frequently than aseptic failures, it constitutes the most devastating complication of joint replacement surgery due to its related high morbidity rates, prolonged hospitalization, and a high risk of complications leading to additional surgery and/or antimicrobial treatment and potential disability [5,6]. The pathogenesis of PJI is related to microorganisms growing in biofilms, which render these infections difficult to diagnose and eradicate. Despite the use of well-established diagnostic methods, a considerable number of PJIs are either found to be culture-negative or misjudged as aseptic failures [7,8,9]. Misinterpretation may lead to wrong or needless antimicrobial treatment, or even to unnecessary surgery [10,11]. 

A correct diagnosis, including the identification of the pathogens and their antimicrobial susceptibility, remains the first step towards successful treatment [12]. Classic microbiological cultures having probably reached their maximum effectiveness, an optimal blend of laboratory, histopathology, and imaging studies, combined with non-culture microbiological methods, is necessary to improve the diagnosis of PJI [13,14]. Implementing an early antimicrobial therapy or planning an appropriate surgical treatment requires an accurate diagnosis of infection. New diagnostic methods have been developed for detecting PJI. Next-generation sequencing (NGS) has recently emerged as a very new and promising technology, which represents a great step forward towards accurate microbiological diagnosis of infectious diseases [2,14]. NGS will probably revolutionize microbiology departments, just as matrix-assisted laser desorption ionization-time of flight mass spectrometry (MALDI-TOF MS) marked a turning point in the identification of microorganisms. NGS platforms apply different approaches that are bound to change the diagnosis (description of new microbiomes), treatment (genotypic detection of resistance genes and virulence factors), and epidemiological analysis (possibility to compare entire genomes). Microbiology is key for the correct diagnosis and management of PJI. It is therefore important to review the usefulness of NGS as far as PJI is concerned. Only few groups have, to date, evaluated the role of NGS in the diagnosis of PJI and the methodologies applied have been far from homogeneous. This review sets out to describe the most important aspects of the microbiological diagnosis of PJI, focusing particularly on the role of NGS.

## 2. Key Aspects about the Microbiological Diagnosis of PJI

### 2.1. Specimens: Type, Location and Number of Samples

Periprosthetic tissue samples provide accurate specimens for detecting infecting microorganism(s), with a sensitivity of 65% to 95% [11,15]. At least three to five periprosthetic tissue specimens from the area in contact with the implant should be sampled [7,16]. A lower number of samples may create interpretation difficulties, while a higher number usually leads to extra costs for the microbiology laboratory.

All explanted components of PJIs, being associated with the formation of biofilms, (mobile parts included), should be sent to a microbiology laboratory [7]. Swabs have a low sensitivity and should be avoided [11]. Microorganisms isolated from sinus tracts usually represent the microbial colonization of skin, rather than the pathogen of infection, so culture of the sinus tract should be avoided [17,18].

Synovial fluid should ideally be sent to the microbiology laboratory in native vials. With regard to the detection of the infecting microorganism, the sensitivity of the synovial fluid inoculated into blood culture bottles is higher than that of traditional cultures [19,20]. Synovial fluid is usually the only sample available for the preoperative microbiological diagnosis of PJI [21]. Blood cultures should always be collected (two pairs) in cases of fever or chills to detect a potential bloodstream infection [11,21].

### 2.2. Type of Recipients

Tissue biopsies, synovial fluid, bone, and other microbiological specimens should be placed in sterile containers and sent to the microbiology laboratory as fast as possible, making sure they are correctly identified, numbered, and at room temperature [22]. Moreover, periprosthetic tissue samples should be collected in different containers in order to allow proper culture interpretation [23,24].

As sonication requires multiple processing steps, which makes the process prone to contamination, the explanted prosthetic components should be collected all together in an air-tight container [7,15,25]. It must be taken into consideration that the sonication of explanted implants in bags is associated with a risk of contamination due to bag leakage and subsequent contamination, particularly with non-fermented Gram-negative bacilli [26].

### 2.3. Etiopathogenesis

PJI can present as acute or chronic. Acute PJI occurs either as an early postoperative or hematogenous infection, whereas chronic infections are caused by low-virulence microorganisms such as coagulase-negative staphylococci or *Cutibacterium* spp., and typically present three months after surgery. Clinical signs of early infection include persisting local pain, erythema, edema, impaired wound healing, hematoma, and fever. Delayed infections may sometimes occur with persisting or increasing joint pain and early prosthetic loosening, but no clinical signs of infection [27]. Such infections are therefore often difficult to distinguish from aseptic failure [28]. Late infections present either with a sudden onset of systemic symptoms (in about 30% of cases) or as subacute infections following unrecognized bacteremia (in about 70% of cases) [7,29].

### 2.4. Previous Antimicrobial Treatment

As the sensitivity of all cultures is reduced in patients receiving antimicrobial therapy [7], antimicrobial therapy should be discontinued at least two weeks prior to collecting specimens, wherever possible. Despite a clear suspicion of PJI, the infecting pathogen is not always successfully isolated by cultures, some authors suggesting that prophylactic antibiotics could interfere with the identification of the pathogen [21]. Preoperative antibiotic prophylaxis does not affect intraoperative cultures in cases of suspected PJI [30,31,32]. It is therefore crucial to administer antibiotic prophylaxis to any patient where a prosthesis is to be implanted in order to protect the prosthesis from infection [33].

### 2.5. Incubation Time

A prolonged incubation time of two weeks is often recommended for diagnosing PJI, especially in the case of chronic PJI cultures [34]. Some investigators have suggested that culture plates may be contaminated during the sampling procedure and/or by prolonged plate incubation time [35]. However, following some basic microbiological recommendations, such as performing the procedures in sterile conditions or following the criteria for culture positivity discussed in Section 2.7, below [26,36], may keep the contamination at manageable levels, even if the plates are incubated for up to 2 weeks [15]. It is, however, possible to shorten the time of positivity (and consequently of incubation) by inoculating sonication fluid in blood culture bottles [37,38,39]. This, however, typically increases the risk of laboratory contamination during bottle inoculation [40]. 

Microbiologists should incubate and look after plates until the end of the prolonged incubation period and should provide clinicians with a preliminary report after 5–7 days of incubation [41]. Clinicians should keep in mind the preliminary nature of this report and that definitive data will not be available before the end of the prolonged incubation period [42].

### 2.6. Recognition of Small Colony Variants (SCV)

The reading of the plates involves the identification of different colonial phenotypes, such as the different morphotypes including SCVs [43,44]. SCVs are a slow-growing subpopulation of bacteria with various phenotypic and pathogenic characteristics [45,46]. This subpopulation of bacteria presents with a slow growth rate, an atypical colony morphology and unusual biochemical characteristics, making bacterial identification challenging for clinical microbiologists [47]. Peculiarly, if bacteria are passaged on culture media, most variants revert to a normal phenotype [48]. Clinically, SCVs are able to persist intracellularly in cells and are less susceptible to antibiotics than their wild-type counterparts, causing latent or recurrent infections [49]. It is important to actively search for SCVs, especially in chronic or recurrent infections caused by virulent microorganisms such as *Staphylococcus aureus* or Gram-negative bacilli. The detection of SCVs should be reported to the clinician because it is crucial to remove all of the biomaterial and administer a combination of antibiotics with intracellular activity over the long term [50,51]. 

### 2.7. Culture Interpretation

Two or more positive periprosthetic cultures constitute unequivocal evidence of PJI, as does as a single positive tissue culture that yields a highly virulent microorganism. In contrast, if a low virulence microorganism grows only in one tissue culture, it should be evaluated in the context of other available evidence, using diagnostic techniques such as sonication fluid culture [21,52]. 

Of all the different sonication protocols, the most widely used one for dislodging bacteria from foreign bodies is based on 1 min or 5 min sonication with or without the use of centrifugation as a concentration process. The suggested positivity cutoff when a non-concentration technique is applied is ≤50 colony-forming units/ml (CFU/mL). When a concentration technique is applied, the suggested cutoff for confirming an infection is ≥200 CFU/mL [53]. The sensitivity and specificity of sonication for the diagnosis of PJIs may be influenced by parameters such as incubation time, previous antibiotic therapy, type of infection (acute or chronic), and CFU cutoff [15]. Sonication fluid culture plates should be inspected daily for microbial growth. If growth is detected, the number of CFUs of each distinctive morphology should be reported according to the standard culture interpretation criteria (Table 1). The sonication fluid culture cutoff depends on the protocol used. Densities ≥50 CFU/mL are usually significant, whereas the significance of those below 50 CFU/mL usually depends on the patient’s clinical situation [53]. If the culture is positive only after enrichment, the result is usually not relevant, the culture being likely to have been contaminated during retrieval, transportation, or processing. Indeed, cases of anaerobe growth, patients receiving antimicrobial therapy, and acute PJI require the implementation of additional diagnostic methods, such as periprosthetic tissue cultures, synovial fluid cultures, and histopathology. 

The sensitivity of sonication fluid cultures is higher in chronic PJI than in acute PJI [36]. This may be due to one of two reasons: (a) in chronic infection, the biofilm is usually more mature and presents with more layers of bacteria than in acute infection, which makes it more difficult to dislodge without sonication procedures [53,54]; or (b) the components explanted following acute infections are typically mobile components (inlays), which have smaller surface areas than total prostheses [55].

The vortexing–sonication procedure is more efficient for biofilm removal than vortexing alone. However, only vortexing with a cutoff of >1 CFU/mL has enough sensitivity, especially in cases of acute PJI [36]. Therefore, vortexing alone may be used in the diagnosis of PJI in a removed prosthesis when sonication is not available. 

Sonication, which involves the physical removal of bacterial biofilms, has been shown to improve the microbiological diagnosis of PJI. Other approaches exist for biofilm dislodgement such as the addition of chemical agents (DTT), although their efficacy is variable [42,53,56]. While some authors claim that the addition of DTT makes the sonication instrument unnecessary, others point out that adding reducing agents has toxic effects on bacterial growth, which may induce a considerable rate of false negatives. 

### 2.8. Multidisciplinary Diagnosis

Various specialties are involved in the management of PJI, each of them contributing their own perspective. These include orthopedic surgery, infectiology, and microbiology. Clinicians usually find it extremely useful for the microbiology laboratory to provide them with a preliminary report after five days of incubation, and a final report at the end of the incubation period [11,57]. When a prosthesis failed early (within the first two years from implantation), the odds that the failure was due to infection are around 70%, as compared with a 16% probability that the cause was aseptic loosening [36]. This emphasizes the importance of systematically searching for infection in all early failures (within the first years from implantation), despite the absence of the classic clinical or laboratory findings suggestive of infection [58].

### 2.9. Non-Culture-Based Methods

There are also non-culture-based methods, especially useful for patients on antibiotics whose cultures have tested negative. Such non-culture-based methods include polymerase chain reaction techniques (PCR) and next-generation sequencing (NGS) [59,60]. The combination of methods such as sonication fluid culture and PCR has shown higher sensitivity and specificity than the application of PCR on its own for pathogen diagnosis [61,62]. MALDI-TOF MS has become indispensable in the identification of pathogens isolated from cultures [14]. Some authors have evaluated the eligibility of MALDI-TOF MS protocols for the direct identification of microorganisms from positive blood cultures, synovial fluid, or even tissue samples, finding optimal concordance with conventional methods [63,64,65]. Our group assessed the diagnostic performance of direct MALDI-TOF MS identification of pathogens from sonication fluid inoculated into blood culture bottles and compared it with intraoperative tissues and conventional sonication fluid cultures. The sensitivity of this approach was 69%, considerably higher than that of conventional sonication fluid (64%, *p* > 0.05) or intraoperative tissue cultures (53%, *p* < 0.01) (Portillo ME, unpublished data). The main advantage of this methodology is the speed at which it can provide microbiologic information by removing a culture step.

#### 2.9.1. Polymerase Chain Reaction Techniques (PCR)

The value of PCR has been extensively studied in the diagnosis of PJI, but the extraction of pathogen DNA from bone or periprosthetic tissue samples remains challenging [66,67] (Table 2). Disruption of the biofilm is an essential step to release the DNA, thereby improving the sensitivity of PCR, especially broad-range PCR [36].

All pathogens, even if unknown, can be identified using universal primers able to amplify bacterial or fungal DNA. This procedure must be followed by the identification of the species by sequencing, a technique also called universal or broad-range PCR [66,75,76,77]. In bacteria, species identification is based on the analysis of the 16S rRNA gene sequence. Similarly, for broad-range fungal PCR, primers targeting conserved areas of genes encoding the 18S, 5.8S, and 28S ribosomal subunits are used. The ITS-1 and 2 regions are among these genes, which allow discrimination between the different fungal species [78]. Therefore, broad-range PCR, although less sensitive than targeted or multiplex PCR, allows the identification of microorganisms previously not thought to cause infection [69,79,80]. Moreover, sequence analysis may be uninformative and even misleading, as it could fail to indicate whether a polymicrobial or monomicrobial infection is present. This may occur, for example, as a result of overlapping electropherogram peaks or of the missed detection of minority species. This problem may be mitigated by a more detailed sequence analysis, or through the manipulation of sequencer data using RipSeq software [81]. 

Targeted or specific PCR methods can be developed for any known microorganism and are able to reach high levels of sensitivity. The analysis is typically performed in real time because the amplification and detection processes occur simultaneously in the same closed vial. In addition, antimicrobial resistance genes or virulence factors can also be detected [71,76,82]. However, if the gene needs to be sequenced, it is better to perform an agarose gel-based PCR analysis [83]. 

Multiplex PCR is a technique where more than two sets of primers are involved in the process of amplifying various target sequences, allowing simultaneous detection and identification of different genes. The main advantage of these systems is the ability to group different targeted PCRs in a single process, which simplifies the technique, saves time and expense, and shortens diagnostic times [11,84,85]. There are different primer panels, including primers for the low-virulence organisms frequently involved in chronic or delayed PJI, such as *Corynebacterium* spp., *Cutibacterium* spp., or other anaerobes [61,86,87]. The Food and Drug Administration has recently approved a new multiplex PCR panel for the diagnosis of joint infections [74]. The kit detects 39 targets (pathogens and resistance genes) containing some anaerobes including some *Cutibacterium* species, but not *C. acnes*, for instance. This panel also includes yeasts and Gram-positive and Gram-negative microorganisms such as *Kingella kingae*. However, it does not detect coagulase-negative staphylococci, which means that its application will probably be more focused on the diagnosis of acute PJI and septic arthritis. The diagnosis of chronic PJIs, probably the most difficult to diagnose, remains, however, unresolved.

#### 2.9.2. Next-Generation Sequencing Methods (NGS)

NGS is increasingly being used in microbiology laboratories due its reduced cost and the wide availability of NGS analytical tools [88,89,90]. The main challenge lies in interpreting the results [91,92,93]. There are two different kinds of NGS: targeted NGS and shotgun metagenomic NGS [14,94] (Table 3). Targeted NGS is also called 16SrRNA gene-based NGS. 

As described above, the 16S rRNA gene has become the most used region for bacterial identification due to being universally present in all bacteria. Therefore, it is possible to sequence this particular gene instead of the entire genome in order to identify the pathogen. This approach unquestionably allows for cheaper and easier interpretations than shotgun metagenomic sequencing. However, this method is associated with some disadvantages such as its inability to detect antimicrobial resistance genes or virulence factors, the risk of producing false negatives, and a certain bias in the quantification of each species [95,96]. 

On the other hand, the shotgun metagenomic approach detects and quantifies all the DNA present in a sample, which can be compared with reference genome databases to identify pathogens. This strategy, which has revolutionized the study of the microbiome and its relationship with different conditions, may be used for diagnosing unknown or even unculturable or unviable pathogens directly from samples [14,97]. 

Additionally, NGS constitutes a useful tool to characterize outbreaks [98,99,100], track transmission [101,102,103], and predict antimicrobial resistance [104,105,106,107,108,109]. The interpretation of results must be based on strict criteria and an understanding of bioinformatics in order to rule out contaminants and avoid overrepresentation of human host DNA, which may interfere with the microorganisms’ DNA sequences, which are less frequent than in human DNA in terms of amount or proportion. The transformation of raw sequence data into clinically applicable information for PJI treatment requires specialized microbiologists. 

Some research groups have investigated the role of NGS in the diagnosis of PJI (Table 4). NGS could play a role in diagnosing PJI due to its high sensitivity. However, the sensitivity and specificity reported in the literature varies according to the type of sample analyzed, the PJI definition applied, and the NGS approach used. 

The sensitivities reported by different groups vary between 61% and more than 95%. Kildow et al. obtained the lowest sensitivity, applying tNGS to different samples using a commercially-available tNGS assay. The assay included a specimen collection kit in which synovial fluid and tissue and prosthetic swabs had to be co-inoculated into a container. As previously discussed, swab samples are not ideal for diagnosing biofilm-related infections. Flurin et al. and Tarabichi et al. also used tNGS, but applied it to tissues and sonication fluid, which resulted in increased sensitivity. Each group used different tissue processing and sonication procedures, as well as different definitions of PJI, so it is very difficult to compare their results. 

The most significant limitation of this study is associated with the numerous knowledge gaps identified in connection with NGS. There is no clear indication of which samples provide the best diagnostic performance, nor is there a proper description of how they should be processed, how nucleic acids should be extracted, how results should be evaluated, how human DNA is to be depleted, or how to analyze the detected microorganisms, especially if several are found. Our results seem to indicate that NGS, as an increasingly accessible tool, holds significant promise. Nonetheless, further, methodologically-sound, comparative studies are needed in order to draw hard-and-fast conclusions.

While application of NGS used to be prohibitive, its cost has dramatically decreased in recent years, especially following the COVID-19 pandemic [117]. This is due to the fact that numerous microbiology departments now need to sequence the SARS-coronavirus in their laboratories, which has made NGS more accessible for clinical use. Moreover, new sequencing analysis platforms have been developed, which make the technique relatively easy to use, even by microbiologists novice to bioinformatics [118].

One of the criticisms that can be levelled at molecular techniques is their predisposition to identify multiple bacteria in cases of both septic and aseptic failure [119,120,121]. Namdari et al. proposed a series of modified diagnostic criteria for PJI, which includes NGS [122]. As a result, as NGS is now a diagnostic criterion for PJI, the significance of the results obtained are greatly strengthened. However, considering that NGS generates thousands of individual sequences, providing information on the organisms living in the joint (microbiota) [122,123], further data regarding the normal joint microbiota are essential to determine which organisms are commensal and which are pathogenic. 

In a nutshell, NGS is a potential diagnostic tool for PJI diagnosis, especially in culture-negative cases [124,125]. NGS can safely be considered a revolutionary technology and the next few years are likely to see its use spread across microbiology services the world over.

## 3. Conclusions and Future Directions

PJI should be suspected in patients with a sinus tract, persistent wound drainage from a joint prosthesis, or a painful prosthesis, particularly in the first few years following implantation [28,119]. 

The management of PJI can, nowadays, rely on new, more accurate, and faster diagnostic techniques. However, samples should always be obtained for culture so that the susceptibility of the pathogen(s) involved can be tested. Non-culture techniques are a valuable supplemental tool in patients with culture-negative PJI caused by fastidious or slow-growing microorganisms and in patients who have previously been on antibiotics. This will allow earlier and more effective treatment. 

Close collaboration between all medical and surgical specialists involved is essential for the correct diagnosis of PJI. Due to the multidisciplinary nature of PJI, the management of these infections is a challenge. A correct microbiological diagnosis is crucial for effective treatment and a successful surgical procedure.

## Figures and Tables

**Table 1:** Sonication fluid culture interpretation criteria.

CFU/mL *	Enrichment Broth	Clinical Significance
≥50	Positive	Yes
<50	Positive	No, except for anaerobes, patients receiving antimicrobials or acute PJI
0	Positive	No, except for anaerobes or patients receiving antimicrobials

* Sonication procedure without a concentration step.

**Table 2:** Different types of PCR applied to the diagnosis of PJI.

PCR	General Description	References
Broad-range PCR	Detection of genes universally present in microorganisms (requires a subsequent sequencing step for identification)	[68,69,70]
Targeted PCR	Specific detection of a particular microorganism and/or resistance mechanism	[71,72]
Multiplex PCR	Simultaneous detection of several microorganisms and/or resistance mechanisms by adding primers of interest	[61,73,74]

**Table 3:** Terms used in the area of Next Generation Sequencing.

Technology	Definitions
Next Generation Sequencing (NGS)	High-throughput, massively parallel sequencing of DNA fragments performed independently and simultaneously.
Whole Genome Sequencing (WGS)	Method for analyzing the entire microbial genome.
Targeted NGS directly from specimen	Focuses only on specific regions of interest in the genome. It requires a pre-sequencing DNA preparation step called *target enrichment*, where target DNA sequences are either amplified or captured and then sequenced.
Shotgun metagenomic NGS directly from specimen	All nucleic acids detected directly from patient specimens are sequenced. The method enables evaluation of bacterial diversity and detection of the abundance of microorganisms.

**Table 4:** Comparison of studies on the use of NGS for the diagnosis of PJI.

Reference	Method	Nr of PJIs	Type of Sample	Sensitivity	Specificity
Kildow et al, 2021 [110]	tNGS	116	Synovial fluid and/or swabs	60.9%	89.9%
Flurin et al, 2021 [111]	tNGS	47	Sonication fluid	85%	98%
Cai et al, 2020 [112]	sNGS	44	Periprosthetic tissues	95.45%	90.91%
Yin et al, 2020 [113]	sNGS	15	Synovial fluid	93.3%	90%
Huang et al, 2020 [76]	sNGS	49	Synovial fluid	95.9%	95.2%
Wang et al, 2019 [114]	sNGS	97	Sonication fluid	94%	95%
Ivy et al, 2018 [115]	sNGS	107	Synovial fluid	84%	100%
Tarabichi et al, 2018 [116]	tNGS	28	Periprosthetic tissues	89.3%	73%
Thoendel et al, 2018 [90]	sNGS	213	Sonication fluid	74.2%	93%
Steet et al, 2017 [89]	sNGS	97	Sonication fluid	88%	88%

Note. tNGS, targeted next-generation sequencing; sNGS, shotgun metagenomic next-generation sequencing.

## Data Availability

Not applicable.
